# A focused, longitudinal analysis of cannabinoid hyperemesis syndrome symptomatology

**DOI:** 10.1186/s12245-021-00367-4

**Published:** 2021-07-29

**Authors:** Philip H. Ma, Katherine M. Joyce, Thayer Morton, David W. Shih, Alexander Weiss, Joseph Miller

**Affiliations:** 1 Wayne State University School of Medicine, Detroit, MI USA; 2 Henry Ford Hospital, Detroit, MI USA; 3 Michigan State University College of Osteopathic Medicine, East Lansing, MI USA; 4 Schulich School of Medicine & Dentistry, London, Ontario Canada

**Keywords:** Cannabinoid hyperemesis syndrome, Cyclic vomiting, Cannabis

To the Editor,

After reading the review article titled “The emergency department care of the cannabis and synthetic cannabinoid patient: a narrative review” by Kevin Takakuwa and Raquel Schears published in the *International Journal of Emergency Medicine* (2021 Feb 10;14(1):10), we want to first congratulate the authors for the successful publication of this article. Additionally, we hope to contribute some of our findings to further enhance this line of research.

## Introduction

As the legalization of cannabis continues to spread in North America, it has become increasingly important for physicians to recognize and treat cannabinoid-related syndromes. In this secondary analysis of a randomized controlled trial that studied the effect of topical capsaicin on patients presenting to the emergency department (ED) with cannabinoid hyperemesis syndrome (CHS), we describe symptomatology and return visits in patients suffering from CHS [1].

## Results

Our study population consisted of 29 participants with a mean age of 30 years. Most patients were Black (90%) and hypertension was the most frequent comorbid condition (28%). All participants experienced nausea and vomiting. Other reported symptoms included abdominal pain (90%), chills (52%), and diarrhea (27%). Abdominal pain was mild (mean visual analog scale (VAS) 2.7 ± 1.2 cm) compared to nausea (mean VAS 7.3 ±2.5 cm). Sixteen patients accounted for 41 repeat ED visits and 10 hospitalizations for CHS-related symptoms within 6 months.

## Discussion

Data indicates that the increased accessibility of cannabis in the USA is associated with an increase in cannabis-related conditions presenting to the ED [2]. Many of these conditions have only recently been characterized and a clearer understanding of CHS symptomatology may improve its accurate recognition and treatment.

While the majority of published studies of CHS obtain retrospective data or present small case series, this prospective data provides an accurate assessment of patient symptoms and high rates of return visits. It also includes data on a largely Black cohort, whereas most published literature on CHS is inclusive of White patients. We present quantified data on the degree of nausea common among CHS patients and the degree of abdominal pain. Contrary to prior studies, we found that abdominal pain severity is overall mild to moderate, especially when compared to nausea severity [3]. Given these findings, a focus on antiemetic treatment rather than analgesics appears warranted in the initial management of these patients.

## Conclusions

In this cohort of CHS patients, it was found that nausea was a larger concern for patients than abdominal pain and that repeat encounters for related symptoms were common. As cannabinoid use becomes more mainstream, physicians in all settings should include CHS in their differential when faced with GI symptoms.

## Data Availability

Data is not publically available

## Abbreviations

CHS: Cannabinoid hyperemesis syndrome
VAS: Visual analog scale
CVS: Cyclic vomiting syndrome
